# The Treatment of Gonorrhœa

**Published:** 1886-10-20

**Authors:** Seneca D. Powell

**Affiliations:** Professor of Minor Surgery, New York Post-Graduate Medical School and Hospital


### THE TREATMENT OF GONORRHŒA.

By SENECA D. POWELL, M. D,
**Professor of Minor Surgery, New York Post-Graduate Medical School and Hospital.**

By gonorrhœa I mean any inflammation of the urethral tract which has been produced either by a specific poison, or by the menstrual fluid, or by leucorrhœal discharges, for I know of no way of distinguishing a urethritis which has for its origin either the one or the other of the above causes. And it matters not what the cause of the urethritis may have been, the fact remains that one is as contagious or virulent as the other.

In the first or introductory stage, if I am fortunate to see the patient at that period, I feel moderately sure of giving speedy relief. I begin the treatment by giving a free purgative, preferring those drugs which act upon the lower bowel rather than a saline cathartic. If the patient has not an excessively sensitive stomach, an emulsion of castor oil, combined with a small dose of spirits of turpentine, (ʒ j) acts well and thoroughly empties the entire tract. I also order two or three drachms of the bicarbonate of soda in Vichy, to be taken in twenty-four hours. Even at this early stage I have found great benefit result from frequently bathing the penis in very hot water. As an injection, a weak solution of the salicylate of soda, two to five grains to the ounce, is used; but more frequently injections of hot water without any medication is preferable.

Injections should be hot.

Latterly, I have aborted gonorrhœal attacks in the first stage in the following manner: After washing the urethra thoroughly with Harrison's urethral syringe, I introduce a rubber canula down below the seat of inflammation, and as I gradually withdraw it, fill the urethra with a dry powder made up of ʒ j resorcin and ℥ j boracic acid, which is allowed to dissolve in situ. I repeat this each day if there be any discharge, but so far never have used it oftener than three times. If the urethra is comparatively dry the day after its application, a weak solution of sulphate of zinc, one grain to an ounce of hot water, is frequently used as an injection.

The patient is ordered to remain quiet, and if possible in bed, while the diet is cut down to milk and mush. The syringe which gives the most satisfaction is the small rubber syringe known as No. 1 *a*, and it is always best to have your patient thoroughly understand the proper manner of using it, for I find very few who are proficient in this detail, although they, as a rule, claim to know just how it should be done. I prefer this style of syringe for several reasons:

The nib or point is very short, and no injury to the sensitive and inflamed mucous membrane can result from its use; and, again, the capacity is small, and there is less likelihood of the secretions being driven back into the urethra by a large volume of water. An injection ought always to follow urination if possible. Large quantities of Vichy or other waters should be taken, not only to dilute the urine, but also to facilitate the more frequent use of the syringe after urination.

The second or inflammatory stage follows quickly upon the first if we have been unsuccessful in aborting the disease. In this stage we should be extremely careful not to attempt too much, for I am positive that many of the cases which have come under my notice have been exaggerated, and much serious damage has resulted from unjustifiable interference by the patient, under the instruction of those whom he has consulted. If a patient comes to me with his penis swollen and engorged with inflammatory products, the lymphatics inflamed, and the glands in the groins painful and swollen, I make no effort at medication by the syringe, but treat the inflammation locally and constitutionally as I would were it in any other part of the body.

There is always more or less increase of temperature and quickening of the pulse, and I begin my treatment by giving the tincture of aconite, in 2 or 3 drop doses, combined with liq. ammon. acetatis, 1 to 4 drachms every two, three or four hours, as indicated. The penis is frequently immersed in hot water or wrapped in borated cotton, and kept wet with lead and opium wash. The amount of bicarbonate of soda and alkaline waters is increased, and the bowels relaxed with mercurial purgative. Just here let me speak of the use of saline cathartics. For several years I have avoided them religiously, for this reason: If there be extensive inflammation, and it goes well back into the urethra near to the neck of the bladder, the mucous membranee being very much thickened and the calibre of the canal lessened, there is, as a rule, more or less spasmodic retention of urine, and the administration of any saline cathartic will, in a great number of cases, increase this difficulty. I have seen this not once, but many times. Be as severe in your restrictions as possible, confining your patient to his bed, if need be, and adhere firmly to your low diet. All exercise should be forbidden wherever possible. If it be absolutely necessary that your patient attend his usual duties, a well adjusted support for the testicles should be ordered. Any further interference in this stage of the disease is, in my opinion, injurious, and especially would I avoid copabia. It is not only useless, but I am positively certain, harmful—increasing the discharge, the ardor urinæ, and the painful erections; occasionally causing a very extensive and persistent rash, to say nothing of its effects upon an irritable stomach. When complications arise, one must be governed by circumstances. Usually the inflammation is modified in three to five days, the discharge decreases and becomes thicker in consistency, the color being whiter, the scalding upon urinating is gone, and the disease enters into the third stage or stage of subsidence.

A physician's assistance is oftener sought at this stage than in the first or second, as its period of duration is very much longer, and may extend over many months and even years; as in a case which recently came under my care, the discharge having lasted four years. Not until after all inflammation has subsided should we use injections otherwise than as I have cited. My first recipe, upon seeing a patient in this stage of the disease, is a good cathartic; and I usually select something mild and which can be repeated every day if necessary, such as rhei and soda, or compound liquorice, pulverized. I also direct the following injection to be used every two or three hours, if convenient:

℞. Zinc sulph .................................. gr. viii,

Morph. sulph ................................ gr. iss,

Sodii bicarb ................................ ʒ ss to ʒ j,

Water ....................................... ℥

I restrict his diet to the plainest foods. No seasoning or condiments allowed. Coffee and tea only in moderate amount and very weak. All kinds of liquors stopped, unless my patient is an habitual drinker and is very much dependent upon his daily dram for his usual appetite and digestion. Very moderate exercise is allowable; but the use of tobacco is entirely, or nearly so, prohibited. I see my patient within the twenty-four hours, and if there be increase in the discharge or change in its character, and there are no evidences of increased inflammation, I begin the use of copaiba; and this I consider the only period wherein it is admissible. If the second stage has lasted any length of time, I much prefer cubebs, given in powder in ʒ ss to ʒ j doses, three or four times a day. In other words, if the mucous membrane is unchanged from frequent attacks of clap or prolonged chronic inflammation, cubebs gives the best results. I have tried about every drug suitable for an injection, and believe that sulphate of zinc ranks them all. Next in my estimation is tannic acid. I never use nitrate of silver in any form for an injection. It has proven unsatisfactory in my hands so often, that I have entirely discarded it. Injections should not be strong enough to cause any pain, and are not only for their astringent effects, but to keep the urethra clean—this being a very important adjunct, in my judgment. The lacunæ, especially the larger ones near the meatus, frequently give us a great deal of trouble by acting as pockets or hiding-places for the disease, and time after time it will spring up after ceasing the use of the syringe. I have in many cases passed a canula and rod armed with cotton saturated with the resorcin and boracic acid, as given before, and wiping the urethra thoroughly in its whole pendulous portion. The small granulations which are sometimes present are more rapidly removed in this way than even by the use of the sound.

I do not mean to imply, gentlemen, that this method of treatment is infallible, but I do say that it has given me more satisfaction and more rapid recoveries than any other.—*College and Clinical Record.*

